# Differentiation of Epileptic Brain Abnormalities among Neurological Patients at Taif Region Using MRI

**DOI:** 10.1155/2023/8783446

**Published:** 2023-11-11

**Authors:** Nahla L. Faizo, Amani A. Alrehaili

**Affiliations:** ^1^Department of Radiological Sciences, College of Applied Medical Sciences, Taif University, P.O. Box 11099, Taif 21944, Saudi Arabia; ^2^Department of Clinical Laboratory Sciences, College of Applied Medical Sciences, Taif University, P.O. Box 11099, Taif 21944, Saudi Arabia

## Abstract

This study was conducted to assess the prevalence of epilepsy among different age groups and gender of neurological patients in the Taif region and define the most common brain lesion, affecting epileptic patients living in the Taif city using MRI. Data from 150 patients who were clinically diagnosed with epilepsy and had brain MRIs were analyzed using SPSS. Statistical significance was considered when the *p* value is 0.05. The percentage of epilepsy was generally higher in males than in females in the Taif city, and seizures were different between the studied age groups. However, epilepsy was more pronounced in females than in males at certain age groups. Moreover, white matter lesions were most commonly found in the studied group (27.7%), followed by focal lesions, edema, and stroke with equal percentages (16.9%) and less commonly with congenital diseases (12%) and atrophic changes (9.6%). Epilepsy was more pronounced in females than in males at certain age groups. White matter lesions were identified as the most common lesion, presenting in epilepsy patients in the Taif city.

## 1. Introduction

Epilepsy is considered as a common and serious neurological disease [1, 2]. Epilepsy disorders are manifested by recurrent and unpredictable seizures, which could be in the form of involuntary movement that may involve part of the body (localized) or the entire body (which are usually generalized), or a change in the function, sensation, behavior, or consciousness level [3, 4]. About 50 million people globally [5] are affected by epilepsy, and the mortality rate in those patients is significantly greater than in the general population. In particular, the prevalence rate was 6,54/1000 in the population with active epilepsy in Saudi Arabia [6]. The rise in mortality of epileptic patients could be linked directly to the primary triggering lesion for the disease (e.g., tumors and brain malformation), to the occurrence of seizures causing sudden, unexpected death, or an indirect relation to seizures resulting in incidents such as road traffic accidents, severe head injuries, and stroke that restrict the amount of oxygen to the brain certain, and genetic syndromes [7, 8]. The consequences of epilepsy extend beyond health complications. Epileptic patients often suffer from psychosocial impairments that drastically worsen their quality of life [9, 10]. Thus, studying the prevalence of epilepsy among a specific population is valuable in understanding the frequency of the disease and the etiologies of epilepsy, as different populations have different medical challenges and requirements.

The initial diagnosing steps for epilepsy are mainly based on the clinical history of seizures validated by irregular electroencephalogram (EEG) electrical discharges in the brain [11]. EEG is helpful to diagnose and distinguish between local and widespread types of seizures; however, it does not show the lesion causing seizures. Therefore, imaging the brain of epileptic patients is important for identifying the abnormal brain area to determine a suitable treatment plan either by therapeutic antiepileptic therapy or the operational elimination of epileptogenic attention [12, 13].

Magnetic resonance imaging (MRI) is an imaging technique suitable for investigating patients with epilepsy [14]. MRI has a noninvasive approach that detects soft-tissue disorders compared to computer tomography (CT) [15, 16]. The disadvantages of MRI, however, are that it is not available in many healthcare centers as it is an expensive device, as well as it requires a longer scanning time compared to other imaging modalities. In patients with epilepsy, the sensitivity of MRI is high in the detection of abnormalities. The role of MRI in epilepsy is well-known in many centers to improve sensitivity and the level of detail [17, 18]. Most of the MR abnormalities are recognized in patients with refractory focal epilepsy and are less likely to be found in patients with one unprovoked seizure [16]. In localized epilepsy, apparent lesions could be found in any of the cerebral brain lobes, most commonly the temporal lobe [13]. Other lesions might be found in MRI, and a possible cause of seizures is an arachnoid cyst [19]. In generalized epilepsy, there were no significant MRI findings [20]; however, sometimes, pathology is found such as stroke, congenital brain disease, white matter, and cerebral edema [21].

More than 70% of epileptic patients are controlled with antiepileptic drugs AEDS, whereas 30–40% of affected patients have a poor response to these medications, and it is called refractory epilepsy regardless of the efforts to find an efficient combination of AEDs [22]. The difficulty in treatment is due to several causes or risk factors causing seizures and many complications and consequences of seizures on the epileptic brain such as lesions, atrophy, cerebral edema, and congenital diseases [23].

There is a lack of country- and region-specific evidence linking epileptic patients and neuropathological abnormalities. In addition, it is still unknown if the presence of neuropathological changes such as white matter lesions, atrophy, or cerebral edema extending beyond the epileptic lesion induces or is the consequence of seizures, which requires elucidation [24–26].

Thus, the current study aimed to assess the prevalence of epilepsy among different age groups and gender of patients attending neurological clinics and hospital wards in the Taif region and define the most common brain lesion, affecting epileptic patients living in the Taif city using MRI.

## 2. Materials and Methods

This retrospective study was conducted at three different Taif hospitals including King Abdulaziz Specialist Hospital, King Feisal Hospital, and Alhada Armed Forces Hospital. 500 medical records were reviewed from the neurology department clinics and only 154 cases had the final diagnosis of epilepsy, but only 150 cases were included in this study. Patients who were included in this study were chosen according to the following inclusion criteria: (1) patients diagnosed with epilepsy whether generalized or localized based on the clinical symptoms and signs of epilepsy. (2) Patients were referred for brain MR imaging, whereas exclusion criteria included patients who did not undergo brain MRI. Patients' data were reviewed using the picture archiving and communication system (PACS) program and tables were created with the following information: age, gender, presence of epilepsy, type (localized or generalized), the presence and reports of EEG, and the presence of abnormal MR images. All reviewed patients underwent 1.5T or 3T MRI scanning. Data were revised, coded, entered, tabulated, and analyzed utilizing the common software called Statistical Package for the Social Sciences (SPSS) version 20. The chi square test was used to compare categorical variables, and statistical significance was considered when the *p* value is equal to or less than 0.05. The confidentiality of subjects was maintained in a designed manner during data collection and reporting.

The study included data from 150 patients alive with active epilepsy: 79 males, aged 2–90 years, and 71 females, aged 4–87 years. The age groups we chose were 0–9, 10–19, 20–29, 30–39, 40–49, and 50-older.

Ethics approval to conduct this study was obtained from the Research Committee of the Ministry of Health and Alhada Armed Forces hospitals in the Taif region.

## 3. Results

After reviewing the suspected cases of epilepsy that underwent MRI, abnormal MRI findings were detected in 83 patients (38 females and 45 males), whereas 67 patients (33 females and 34 males) had normal MRI.

The prevalence of epilepsy was estimated among males and females among different age groups. Our results have shown that males are significantly affected more than females in the Taif city in the age groups from 0 to 9 years old, 20 to 29, 40 to 49, and above 50 years old, whereas females were more affected in the age groups from 10 to 19 and 30 to 39 years (Table 1).

**Figure 1:**
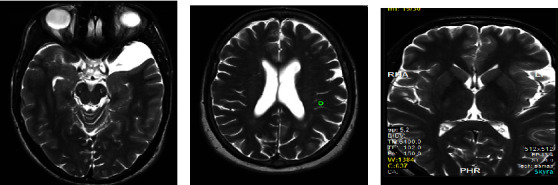
The type of lesion presented in MR images. (a) Axial T2W lesion was seen in the left temporal region; mostly, it is an arachnid cyst. (b) Axial T2 WI shows a prominent ventricular system and edema. (c) Axial T1 and T2 in the left parietal region suggestive of old stroke.

When examining the brain MR reports by the radiologist, six main conditions or lesions were recorded including focal lesions (such as arachnoid cyst in Figure 1(a)), regional atrophy, edema (Figure 1(b)), stroke (Figure 1(c)), white matter, and focal lesions. The prevalence of brain lesions presented on MR images of the brain showed that the most common lesion causing epilepsy in neurological patients living in the Taif city was white matter lesions with a significant *p* value of 0.001, as shown in Table 2.

**Figure 2:**
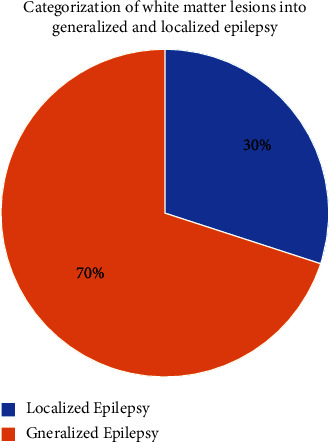
Categorization of white matter lesions into generalized and localized epilepsy. The figure demonstrates that most patients with white matter lesions were diagnosed with generalized epilepsy.

Afterward, the categorization of patients with the commonly identified lesion into focal and generalized epilepsy was performed. We classified patients with white matter lesions into generalized and localized epilepsy according to their clinical reports. Results showed that patients with white matter lesions who were diagnosed with generalized epilepsy were more than the ones with localized type, as shown in Figure 2.

## 4. Discussion

The purposes of this study were to assess the prevalence of epilepsy among different age groups and gender of patients in the Taif region and to define the most common brain lesion, affecting epileptic patients living in the Taif city. We have assessed the prevalence of epilepsy in both genders and the distribution of epilepsy disorders among different age groups. Our findings have shown that 52.22% of patients were males and 47.33% of females were affected by epilepsy. However, this distribution was different between the studied age groups. In the age groups from 0 to 9 years old, 20 to 29, 40 to 49, and above 50 years old, the prevalence in males was higher than in females, but the differences were variable. Surprisingly, the prevalence between sexes in the age groups from 10 to 19 and 30 to 39 years revealed that seizures in females are more dominant than in males. According to Saudi customs and tradition, the onset of epilepsy in females at the age of 10–19 years could be the stress derived from the beginning of the cyclic period. However, the epileptic seizure at the age of 30–39 years old is possibly due to the stress associated with the start of taking responsibility for marriage, creating a family, and raising children (Table 2). Regarding men in the Saudi culture, the age range of 20–29 years is the range of starting to take on bigger responsibilities for their families as well as the begin of planning for their own lives and futures.

Considering the types of lesions shown in MR images that are associated with epilepsy, our findings have shown that the causes of epilepsy in suspected patients who underwent MRI of the brain included white matter lesions most commonly (32%), followed by focal lesions, edema, and stroke with equal percentages (19%), and less common were regional atrophic changes (11%).

Despite that our findings have shown that patients with white matter lesions suffer from either generalized or focal types of epilepsy, generalized epilepsy was diagnosed in most patients. Several studies have shown that the cerebral excitability and pathophysiology of generalized epilepsy are different from those of focal types [27], yet other studies have proved that the generalized type frequently starts as focal seizures and then proceeds to generalized epilepsy [28]. This would explain why white matter lesions contribute more to the types of lesions causing epilepsy. Most of the detected focal lesions in this study were related to temporal lobe epilepsy, which is the commonest type among all focal epilepsies [24, 29].

Our findings have also revealed that stroke is one of the main findings in the brain of epileptic patients. Poststroke epilepsies are common among the elderly [30]. In our study, stroke-causing epilepsies were found in all age groups, but 50% were found in patients older than 50 years old. The other 50% of epileptic patients who had a stroke were younger than 50 years old. This could be explained by the presence of stroke risk factors in the Taif city population such as smoking, which doubles the risk of having a stroke [31].

Moreover, regional brain atrophy, which is usually seen in infants, is also a possible cause of epilepsy. Patients with cerebral atrophy suffer from seizures that sometimes could be of the refractive type. However, some studies still have a strong debate about the possibility of recurrent seizures causing brain atrophy [32, 33].

In addition, cerebral edema was one of the findings in the brain MRIs of epileptic patients. Postseizure cerebral edema has been previously described in the literature as an acute brain injury in epileptic patients [34, 35]. However, considering Taif city is a high-altitude region, it is difficult to conclude whether the cerebral edema is due to epilepsy disorder or a consequence of living in a high-altitude region. Cerebral edema is considered a physiological effect of living in a high-altitude region. Although the exact mechanism of this reaction is not clearly understood, it is believed that hypoxia at altitude induces vasodilation which results in brain edema [36]. Thus, at the level of our study, it is difficult to conclude whether cerebral edema is a cause or consequence of seizures since other factors could contribute to the results as cerebral edema. Therefore, it is recommended for future studies to investigate the inference of a causal relationship between the presence of the discovered lesions and epilepsy, since living at high altitudes such as the Taif region is a contributing factor for lesions such as brain edema.

One of the major limitations of this study was that most patients were imaged using the 1.5 T MR system, which might be the cause of not detecting mild brain lesions. Moreover, the hospitals from which the data were collected used different MRI protocols for scanning epilepsy patients.

## 5. Conclusions

This study has demonstrated that epilepsy was generally more pronounced in males; however, females were more affected than males at certain age groups. Furthermore, this study delineated that the most commonly presented lesion in epilepsy patients in the Taif city was white matter disease, and the least common type of lesion detected was regional atrophy.

## Figures and Tables

**Table 1:** Prevalence of epilepsy between males and females.

Age group	Frequency			Percentage		
	Female	Male	Total	Female	Male	Total
0–9	7	9	16	4.67%	6.00%	10.67%
10–19	26	11	37	17.33%	7.33%	24.67%
20–29	6	24	30	4.00%	16.00%	20.00%
30–39	15	12	27	10.00%	8.00%	18.00%
40–49	10	19	29	6.67%	12.67%	19.33%
50 or older	7	4	11	4.67%	2.67%	7.33%
Total	71.00	79.00	150.00	47.33%	52.67%	100.00%
*p* value	0.001					

Epilepsy was significantly higher in males than in females in the Taif city (*p* value = 0.001).

**Table 2:** Most common lesions causing epilepsy in MR images.

Variables	Conditions	Frequency	Percentage (%)
Abnormal condition on MRI	Regional atrophy	8	9.6
	Edema	14	16.9
	Focal lesion	14	16.9
	Stroke	14	16.9
	White matter	23	27.7
	Congenital brain diseases	10	12

Total	6	83	100%

*p* value	0.001		

A high incidence of white matter lesions was found in epileptic patients, compared with other lesions.

## Data Availability

The excel data sheet used to support the findings of this study may be released upon application to the Alhada Armed Forces hospitals and the Ministry of Health hospitals in the Taif regions. Taif region in Saudi Arabia is a military region and therefore, the author should acquire approval for sharing the data of this research. The author who can be contacted at (nfaizo@tu.edu.sa +966558800943).
